# Phytochemical screening, HPLC analysis, antimicrobial and antioxidant effect of *Euphorbia parviflora* L. (Euphorbiaceae Juss.)

**DOI:** 10.1038/s41598-024-55905-w

**Published:** 2024-03-07

**Authors:** Muhammad Adil, Faten Zubair Filimban, Ambrin, Atifa Quddoos, Ayaz Ali Sher, Muhammad Naseer

**Affiliations:** 1 Department of Chemical and Life Sciences, Qurtuba University of Science and Information Technology, Peshawar, Pakistan; 2 Division of Botany, Department of Biological Sciences, Faculty of Science, King Abdulaziz University, Jeddah, Saudi Arabia; 3 Department of Botany, Islamia College, Peshawar, Pakistan; 4 Centre for Plant Sciences and Biodiversity, University of Swat, Charbagh, Pakistan

**Keywords:** Bio-active constituents, Antibacterial activity, Antimicrobial activity, Antioxidant potential, Drug resistance, Natural resource, *Euphorbia parviflora*, Biological techniques, Microbiology, Molecular biology

## Abstract

Plant extracts are actively being used worldwide due to the presence of biologically active constituents helping in the preservation of food, and to aid against various diseases owing to their antimicrobial and antioxidant potential. The present research work was carried out to investigate the phytochemical constituents, antimicrobial activity, and antioxidant activity of different extracted samples of *Euphorbia parviflora*. Anti-microbial studies were carried out by Agar well diffusion while the DPPH method was employed for investigating anti-oxidant activity. Three samples from methanol, chloroform, and ethyl acetate extract were tested against five different bacterial strains comprising two species from Gram-positive bacteria i.e., *Staphylococcus aureus* and *Bacillus subtilis* and three species from Gram-negative bacteria i.e. *Escherichia coli*, *Pseudomonas aeruginosa* and *Klebsiella pneumonia* along two fungal strains i.e. *Candida albicans* and *Aspergillus niger.* The results of the qualitative phytochemical analysis showed that methanolic, chloroformic, and ethylacetate extract of *Euphorbia parviflora* consist of alkaloids, reducing sugars, flavonoids, terpenoids, tannins, and saponins. The total phenol and flavonoid content of *E. parviflora* showed that the methanolic extract of *E. parviflora* had a significantly higher total phenolic content (53.73 ± 0.30 mg of GAE/g) and flavonoid content (44.62 ± 0.38 mg of than other extracts. The content of total phenolic and flavonoids was more in methanolic extract as compared to other extracts of *E. prolifera.* The HPLC analysis showed that in the chloroform extract of *E. parviflora* Cinnamic acid (4.32 ± 2.89 mg/g) was dominant, in methanol extract quercetin (3.42 ± 2.89 mg/g) was dominant and in ethyl acetate extract of *E. parviflora* catechin (4.44 ± 2.89 mg/g) was found dominant. The antimicrobial activity revealed that amongst all the extracts the highest antibacterial activity was shown by methanolic extract against *B. subtilis* and *Staphylococcus aureus* as compared to the other extracts. The antioxidant activity revealed that methanolic extract of *E. parviflora* demonstrated higher antioxidant activity (82.42 ± 0.02) followed by chloroform extract (76.48 ± 0.08) at 150 µg/mL. The aim of this study was primarily to evaluate the potential of this plant as a reliable source of antimicrobials and antioxidants that may be used for the treatment of various infectious diseases in the future. The study provides evidence that this plant can act as a reliable source of antimicrobial and antioxidant agents and might be used against several infectious diseases.

## Introduction

Since the beginning, human beings have been utilizing plants for the development of drugs. Traditional use of natural products in the treatment of diseases has brought to light the importance of searching for new therapeutic drugs^1^ from these plants. The therapeutic importance of plants depends upon the type of phytochemical compounds, they possess, showing various physiological effects on the human body. Hence, the phytochemical screening method can be employed to detect these compounds present in plants which may be used as the basis for modern drug development^1,2^.

There is an increase in the ratio of the resistance of microbes against synthetic drugs and a decrease in the arrival of new antimicrobial medicines. To treat these microbial infections, the focus has been shifted to the attainment of novel, effective but affordable drugs for treatment against microbial infections prevailing especially in developing countries where 50% of the mortality rate is due to infectious diseases. Phytochemicals are the products synthesized as a result of metabolic processes occurring in plants^3^.

Previously, for the treatment of infections-causing agents, various compounds acting as antimicrobials were identified^4^ from natural as well as unnatural resources, but just fewer number of these antimicrobial constituents were affordable worldwide. The advancement in resistance, shown by bacteria, to various drugs, has intensified the problem of affordability as well as accessibility of these drugs throughout the world^5^. Hence, rates of morbidity, mortality, and health have become costly. Therefore, the need to derive antibiotics from non-synthetic products is given utmost importance in modern-day medicines to cope with the socio-economic and health problems occurring due to these resistant varieties of microbes^5^.

Formerly, a vast number of chemical constituents were isolated from natural and synthetic substances to treat or control agents causing infections^6^. Due to the increase in multi-drug resistant bacteria, the accessibility as well as affordability of anti-biotics has become difficult throughout the world^5^. Hence, the effectiveness of the treatment is reduced causing an increase in the rate of mortality, disease occurrence as well as health care cost. Therefore, the need to search for innovative antibiotics from natural resources is highly stressed in the field of modern medicine which might help in overcoming the negative socio-economic as well as health impact caused by multi-drug resistant microbes^6^.

Free radicals play an important role in the occurrence of various diseases. Reactive oxygen species i.e. ROS are produced in the body via utilizing redox enzymes. These ROS are synthesized uninterruptedly by reacting with foreign chemicals in a suitable environment. Under normal circumstances the antioxidants help remove the ROS produced in the human body, maintaining a balance between the production of ROS and the quantity of antioxidants available. Over-production of ROS as compared to the low number of antioxidants results in the development of oxidative stress, thus acting as a potential source of causing illnesses such as diabetes, inflammation, abnormal cell division, and ulcers of various kinds, pacing the process of aging, etc.^7^.

Antioxidants have scavenging capability which negates oxidation in easily oxidizable substrates thus preventing the formation of free radicals^8^. Antioxidants are abundant in plants in forms including polyphenols, ascorbate, terpenoids, and tocopherols. They help the body defend itself against free radicals causing oxidative stress. Although, human has an effective mechanism of defense built it gets weakened with the aging process. Thus, the human body needs to be supplied with food that can act as effective antioxidants. Due to the presence of these free radicals biomolecules such as amino acid chains, fatty acids, and deoxyribonucleic acids are severely damaged^9^.

Genus *Euphorbia* L. is among one of the largest angiospermic plants belonging to the family Euphorbiaceae Juss. consisting of about 2000 species distributed in tropical and sub-tropical climatic regions. It has caught attention earlier worldwide particularly due to its rich morphological nature as well as almost cosmopolitan distribution^2^. *Euphorbia* species are easily recognizable due to the presence of specialized inflorescence and milky latex. They have various chemical constituents such as terpenoids, steroids, glycerol, flavonoids, and chloroacetophenones. Multiple pharmacological activities such as antifungal, anti-viral, anti-inflammatory, and cytotoxic potential have also been reported from *Euphorbia* species^10^.

*Euphorbia parviflora* L. belongs (family Euphorbiaceae Juss.) is an annual herb growing erect up to 15–30 cm in height. Leaves are elliptic to oblong, with obtuse apex and rounded base. Involucres with white or rarely pinkish-red petaloid limbs. Fruit is capsule type; capsules are subglobose and hairy. The seeds are reddish brown. The plant has some medicinal value as it is useful in dysentery and diarrhea. Infusion of dried leaves is an astringent used in dysentery, diarrhea, menorrhagia, and leucorrhea. It contains phenolic substances, essential oils, glycosides, and alkaloids^11^. The main aim of this research is to analyze the phytochemicals and antimicrobial along with the antioxidant activity of *E. parviflora.*

## Materials and methods

### Plant collection

Fresh samples of *Euphorbia parviflora* were collected in April 2022 on private land at Bunir, Khyber Pakhtunkhwa. Permision was obtained from the land owner. The plants were identified by Mr. Ghulam Jellani, at the Department of Botany, University of Peshawar, Pakistan. The plant was preserved on herbarium sheet and voucher number QUSIT424AD was given and deposited in herbarium for future reference. The plants were dried in the shade at room temperature 25 °C for 20 days. Plant parts were grounded to 60 mesh diameter fine powder with the assistance of an electric grinder. 50 g of sample powder was saturated each in 250 mL methanol, chloroform, and ethyl acetate. After resting for three days, the extracts were filtered by Whatman filter paper No. 1823. This process was applied triple time and the extracts were combined and then it was concentrated utilizing a rotary type of evaporator. The obtained extracts were then packaged and stored at around 4 °C.

### Qualitative phytochemical screening

Phytochemical screening of chloroform, methanol, and ethyl acetate extracts of *Euphorbia parviflora* was carried out via standard procedures.

### Saponins

About 200 mg of extract obtained from the plant sample was put into test tubes. A 10 mL distilled water was added to it and boiled. The froth appeared persistent for more than three minutes showing the presence of saponin^12^.

### Alkaloids

About 200 mg of plant extract from each sample was mixed with 2% H_2_SO_4_ and heated for 2 min. After boiling, it was thoroughly filtered and a few drops of Dragendroff’s reagent were mixed in it. The appearance of orange-red precipitate proved the availability of alkaloids^12^.

### Flavonoids test

200 mg of each extract was dissolved in NaOH and then HCl was added to it. Turning of solution into colorless from yellow confirmed the existence of flavonoids^13^.

### Tannins test

Distilled water was mixed up with a small quantity of sample and then boiled. Filtrate was obtained and a few drops of Ferric chloride were added to the filtrate. The appearance of blackish-green color indicated the tannin's availability^13^.

### Glycosides test

NaOH solution was added to the sample extract to neutralize it, then it was hydrolyzed with the HCl. Solution A and B of Fehling solution were added to it which created precipitates in red color indicating the availability of glycosides^14^.

### Reducing sugars test

Distilled water was added to extract from each sample and filtered. Little drops of Fehling’s solution A and B were mixed into the filtrate and then it was boiled for some time. Precipitate in orange-red color appeared showing the reduction of the presence of sugars^13^.

### Terpenoids test

200 mg extract from each sample was mixed with 2 mL of chloroform along with 3 mL fully concentrated H_2_SO_4_ added to it forming a layer. The interface appeared to be reddish-brown which indicated the presence of terpenoids^14^.

### Total phenolic content assay

The total phenolic content of *Euphorbia parviflora* extracts was analyzed following the protocols of^15^ 4.5 mL of distilled H_2_O mixed up gradually to 0.5 mL of extract from the sample. Then 0.2 mL of the Folin–Ciocalteu phenol reagent was added to it. A 0.5 mL of saturated solution of Na_2_CO_3_ was added too. At last, 4.3 mL of distilled water was added to the solution. The reaction mixtures were incubated and kept in the absence of light at room temperature for an hour duration. The absorbance potential was measured at 725 nm. Total phenolic content was expressed in (mg GAE/g) i.e., mg of Gallic acid equivalents (GAE) per gram of dry sample.

### Flavonoid content

Using the colorimetric method of aluminum chloride, the flavonoid constituent of *Euphorbia parviflora* extracts was evaluated following the procedures proposed by^16^. Slowly and gradually, 0.5 mL sample was mixed with 1.5 mL of methanol (95%), 0.1 mL of aluminum chloride (10%), 0.1 mL of 1 M potassium acetate, and 2.8 mL of distilled water. The mixture was then incubated and kept in the dark for half an hour. Finally, absorbance was noted at the range of 415 nm. The flavonoid content was measured in (mg QE/g) i.e. mg quercetin equivalents per gram of the dried sample.

### HPLC analysis

The HPLC analysis of chloroform, methanol, and ethyl acetate extract of *Euphorbia parviflora* was performed with A Shimadzu LC-20AD HPLC system (Shimadzu, Japan) having a delivery system consisting of binary solvent (LC-20AD), an injector of Rheodyne type possessing 20 µL sample loop and DAD detector (SPD-M 20 A). Through the mechanism of reverse phase column chromatographic separation was carried out (Capcell Pack C-18, MGII, 5 µm, 250 mm × 4.6 mm) with an extended guard column. The mobile phase consisted of methanol–acetonitrile water (40:15:45, v/v/v) containing 1.0% acetic acid with isocratic elution for 30 min. Shimadzu LC solution software was applied for acquiring the data and processing. The range of the Diode array detector was kept between 240 to 800 nm. The rate of flow was 1 mL/min and the volume of samples and standard solutions were taken as 20 µL. By keeping track of retention time and analyzing UV spectra the peaks were identified by comparing them with reference standards, confirming them by running the samples with a small amount of the standards.

### Anti-microbial activity

The agar well diffusion process proposed by^17^ was applied to evaluate the antimicrobial potential of *Euphorbia parviflora*. Both Gram-positive and Gram-negative bacterial strains namely *Staphylococcus epidermidis, Bacillus subtilis, Pseudomonas aeruginosa, Escherichia coli, Klebsiella pneumonia,* and two fungal strains *Candida albicans* and *Aspergillus niger* were used. Bacterial and fungal strains were received from the Biotechnology Center of Agricultural University, Abasyn University Peshawar. The strains were initially cultured on nutrient substrate and then were incubated for 24 h before the observations. Nutrient agar was melted and then cooled to about 40 °C before pouring them into sterilized petri dishes. Wells with a gap of 24 mm were built in media utilizing a metal cork borer having a diameter of about 6 mm diameter. Bacterial strains of 4–8 h of age duration were spread over the layer of nutrient agar with the help of a sterilized cotton swab. The process was repeated in three cycles, turning the plate in between each streaking. About 2 mL/1 mg of respective extract, which was dissolved in DMSO was then added to the customized wells. The remaining wells were supplied with standard antibiotics to serve the controlled ones. The prepared plates were incubated for 24 h at 37 °C. These plates were then observed for zones of inhibition.

### DPPH free-radical scavenging assay

Free radical scavenging assay utilizing 1,1-diphenyl-2-picrylhydrazyl radical (DPPH) Sigma-Aldrich of *Euphorbia parviflora* extracts was carried out following the procedures^18^. A 25 mg of dry crude extracts was added in distilled methanol and a 50 mL diluted solution was prepared. This solution serves as a base for the development of various solutions for the test to be performed i.e. 50 μg/mL, 100 μg/mL, 150 μg/mL. Ascorbic acid was taken as a standard drug. In a test tube, about 5 mL of each solution was taken to which 1 mL of 0.001 M of DPPH solution was added up to it. All of the solutions were kept in the absence of light for half an hour. In a controlled solution, a methanol extract of about 5 mL was added to about 1 mL of DPPH. After the completion of the incubation period, the mixtures were observed for the antioxidant activity potential using an Optima UV Visible spectrophotometer at a wavelength of 517 nm. The experiments were performed and observed in triplicate. The percentage of DPPH inhibition was determined by using the formula:

$$(\% )\;{\text{Scavenged }}DPPH = \frac{{{\text{Absorbance of control}} - {\text{Absorbance of sample}}}}{{\text{Absorbance of control}}} \times 100$$

### Institutional guidelines

All the methods were carried out in accordance with relevant Institutional guidelines and regulations.

## Results and discussion

### Qualitative phytochemical screening

Phytochemical screening is used to evaluate the constituents of the plant extracts, and their predomination, along with the search for bioactive constituents that may be helpful in the production of therapeutic drugs^19^. In the current study the qualitative phytochemical analysis of chloroform, methanol, and ethyl acetate extracts of *E. parviflora* is carried out as shown in Table 1. Alkaloids, tannins, Phenols, flavonoids, and terpenoids were detected in all extracts. The therapeutic potential of *E. parviflora* might be due to the presence of these phytochemicals. Terpenoids show multiple pharmacological activities i.e., working as active agents against inflammation, cancer, viruses, and bacteria along with hindering cholesterol synthesis. Flavonoids are known to have antioxidant effects, inhibiting the initiation, promotion, and progression of tumors. Tannins possess antiviral, antibacterial, and antitumor activity^20^. Alkaloids obtained from to beta-carboline group *have strong antimicrobial, anti-HIV, and antiparasitic activities*^21^.

**Table 1:** Phytochemical screening of various extracts of *Euphorbia parviflora.*

Phytochemical compounds	Chloroform extract	Methanol extract	Ethyl acetate extract
Alkaloids	+	+	+
Tannins	+	+	+
Phenols	+	+	+
Glycosides	−	+	+
Reducing sugar	−	−	−
Saponins	+	+	−
Flavonoids	+	+	+
Terpenoids	+	+	+

+ indicates presence, − indicates absence.

### Total phenolic and flavonoid content

Phenolic content and flavonoid content present in natural products are the significant criteria for evaluating the extract quantitatively as well as its biological strength as they play an essential role in overall physiological processes^22,23^. In this study, the quantity of total phenolic and flavonoid content of chloroform, methanol, and ethyl acetate extract of *E. parviflora* is determined as shown in Table 2. The results of total phenolic content showed that the methanolic extract of *E. parviflora* had a significantly higher total phenolic content (53.73 ± 0.30 mg of GAE/g) followed by chloroform extract (46.65 ± 0.54 mg of GAE/g),. Similarly, the analysis of the total flavonoid content, showed that the methanolic extract of *E. parviflora* contained higher total flavonoids (44.62 ± 0.38 mg of QE/g) followed by the ethyl acetate extracts (35.42 ± 0.43 mg of QE/g) this study is in line with the investigation carried out by^23^. The content of total phenolic and flavonoids was more in methanolic extract as compared to other extracts of *E. parviflora.* Results revealed that methanol was more significant than the other two solvents in the extraction of phenols and flavonoids. Similar results were shown by^24^. This could probably be because of the higher polarity and better solubility of methanol for these phytochemicals^24^. Phenolics possess a wide spectrum of biochemical activities such as antioxidant antimutagenic, and anti-carcinogenic as well as the ability to modify gene expression. Flavonoids are active constituents performing various biological activities such as resisting microbial, ulcer, arthritis, angiogenic, and cancerous diseases along with inhibiting the formation of mitochondrial adhesion^24^.

**Table 2:** Total phenolics content, total flavonoids content of the various extract of *Euphorbia parviflora.*

Extracts	Phenol content (mg GAE/g extract)	Flavonoid content (mg quercitin/g extract)
Chloroform	46.65 ± 0.54	30.53 ± 0.46
Methanol	53.73 ± 0.30	44.62 ± 0.38
Ethyl acetate	35.62 ± 0.50	35.42 ± 0.43

### HPLC

**Figure 1:**
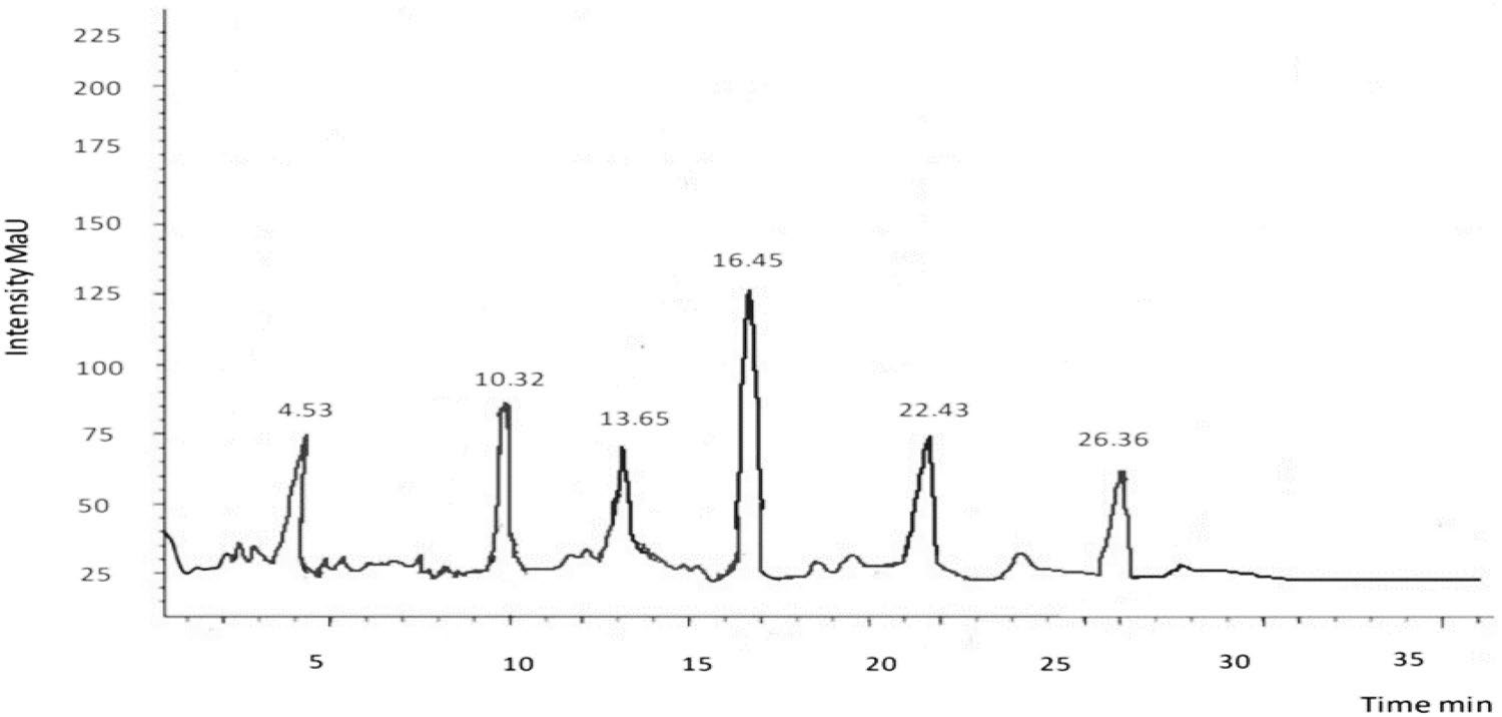
HPLC chromatogram of chloroform extract of *Euphorbia parviflora.*

**Figure 2:**
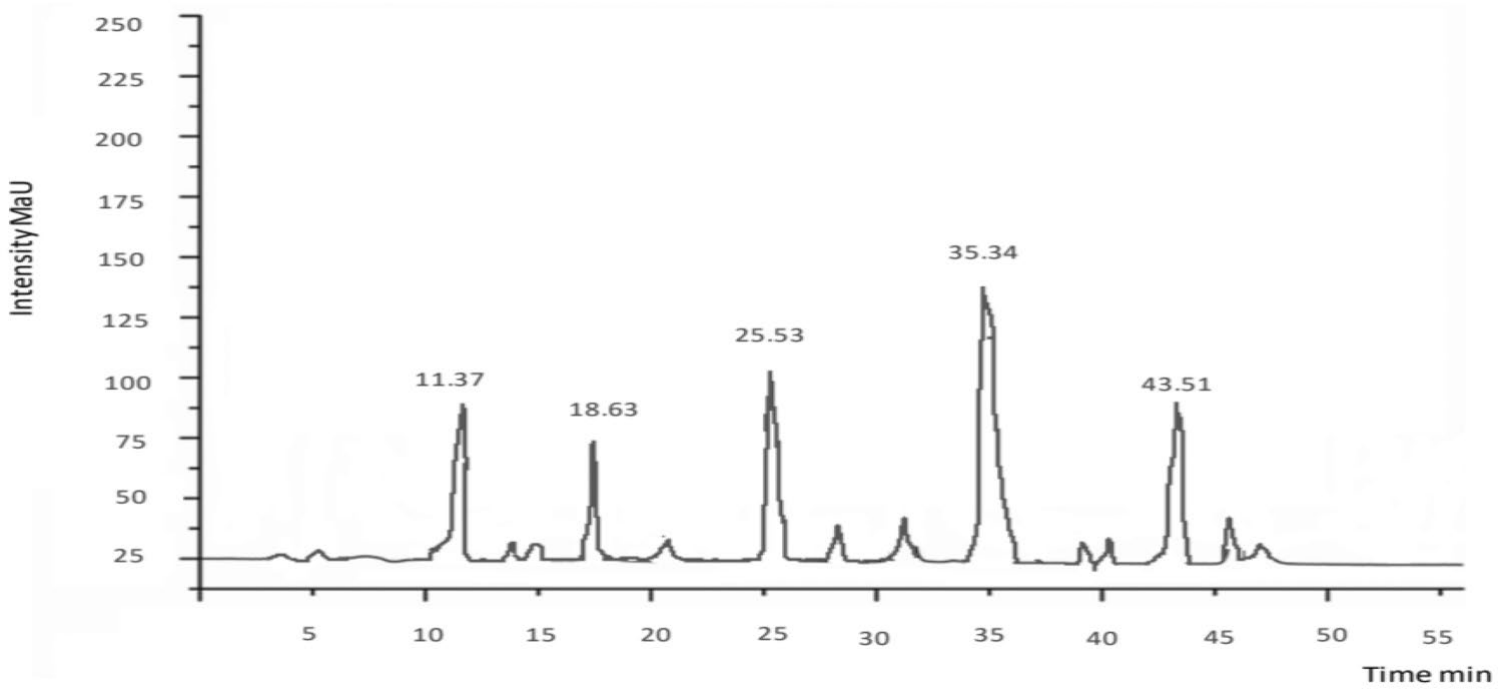
HPLC chromatogram of methanol extract of *Euphorbia parviflora.*

**Figure 3:**
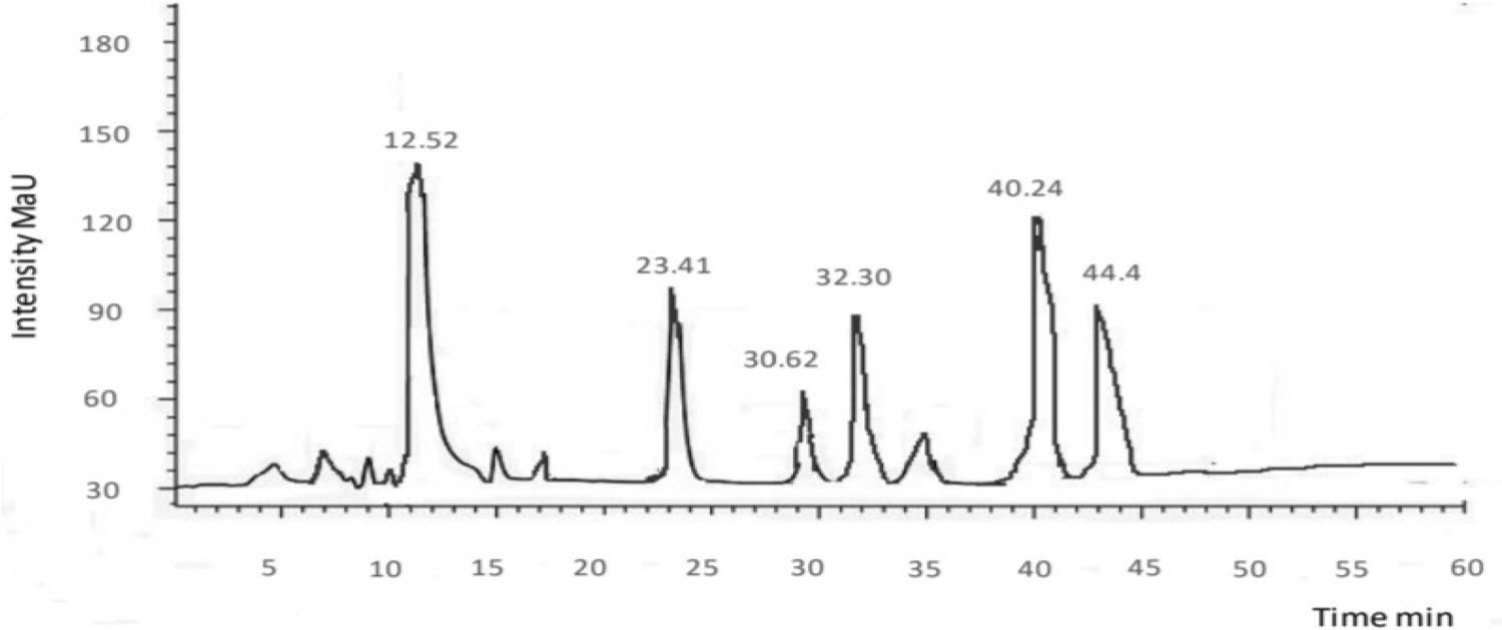
HPLC chromatogram of ethyl acetate extract of *Euphorbia parviflora.*

The HPLC analysis is one of the most frequently applied techniques to evaluate phenolic compounds present in plants. Characterization of every phenolic compound is not possible due to the rich diversity and complexity present in plants. Rather main categories and their representative phenolics could be identified with ease using HPLC technique. To identify the variety of components with antioxidant and antimicrobial activities, an HPLC was carried out to identify and quantify flavonoids and phenolic compounds in chloroform, methanol, and ethyl acetate extract of *E. parviflora.* The HPLC chromatograms of extracts and tested compounds are shown in Figs. 1, 2, 3. Quantification was done through the process of standard calibration. The amount of compound identified in the analyzed samples is shown in Tables 3, 4, 5. In the chloroform extract of *E. parviflora* Cinnamic acid (4.32 ± 2.89 mg/g) was the dominant constituent followed by kaempferol (3.40 ± 0.54 mg/g). Similarly, in the methanol extract of *E. parviflora* quercetin (3.42 ± 2.89 mg/g) was the dominant constituent followed by vanillic acid (2.67 ± 0.54 mg/g). However, in the ethyl acetate extract of *E. parviflora* catechin (4.44 ± 2.89 mg/g) was the dominant constituent followed by luteolin (2.37 ± 0.54 mg/g). All quantified phenols have therapeutic uses i.e., rutin has a significant anticancer, antioxidant, and anti-inflammatory effect and antimicrobial properties. Similarly, kaempferol shows antimicrobial potential, antioxidant, and cytotoxic potential^25^. Quercetin is known for its anti-inflammatory, antihypertensive, vasodilator effects, antiobesity, antihypercholesterolemic, and antiatherosclerotic activities. Luteolin possesses anti-oxidant, anti-inflammatory, anti-bacterial, anti-diabetic, and anti-proliferative potential properties^26^.

**Table 3:** HPLC of chloroform extract of *Euphorbia parviflora.*

Peak no.	Retention time	Phenolic/flavonoids compounds	Quantity (mg/kg)
1	4.53	Kaempferol	4.40
2	10.32	Apigenin	1.54
3	13.65	Cinnamic acid	2.32
4	16.45	Rutin	3.54
5	22.43	5-*p*-Coumaroylquinic acid	2.66
6	26.36	p-coumaric acid	1.14

**Table 4:** HPLC of methanol extract of *Euphorbia parviflora.*

Peak no.	Retention time	Phenolic compounds	Quantity (mg/kg)
1	11.37	Caffeic acid	3.32
2	18.63	Myricetin	1.50
3	25.53	Quercetin	4.35
4	35.34	Vanilic acid	2.37
5	43.51	Naringin	1.66

**Table 5:** HPLC of ethyl acetate extract of *Euphorbia parviflora.*

Peak no.	Retention time	Phenolic compounds	Quantity (mg/kg)
1	12.52	Luteolin	2.37
2	23.41	Cafeic acid	1.31
3	30.62	Ferulic acid	2.14
4	32.30	Catechin	4.44
5	40.24	Chlorogenic acid	0.56
6	44.48	Isoquercitrin	1.49

### Antioxidant activity

The scavenging of free radicals through the DPPH is an approved technique for determining the antioxidant activity of extracts taken from plants. This method requires less time for analysis and, hence, is used widely for measuring the anti-oxidant potential of plant extracts. Due to their hydrogen-donating ability, DPPH is considered to be well effective antioxidant. Removal of these free radicals is vital to inhibit the toxic role of these in various diseases, including cancer^26,27^. The results of the antioxidant activity of chloroform, methanol, and ethyl acetate extracts of *Euphorbia parviflora* are shown in Table 6. The results revealed that methanolic extract of *E. parviflora* demonstrated higher antioxidant activity (82.42 ± 0.02) followed by chloroform extract (76.48 ± 0.08) at 150 µg/mL. The antioxidant activity of *E. parviflora* extracts showed that it depends on the amount of dose given (Table 6). The standard (l-ascorbic acid) exhibited significantly higher DPPH radical scavenging activities than the DPPH radical scavenging activities of *E. parviflora* extracts. The lowest antioxidant activity (61.42 ± 0.02) was shown by ethyl acetate extract at 150 µg/mL. The concentrations of the studied sample extracts required to scavenge 50% of the DPPH radicals (IC_50_) were also determined in this study. The IC_50_ values for the chloroform, methanol, and ethyl acetate extract were 64.54 µg/mL, 46.6 µg/mL, and 83.72 µg/mL respectively. However, the IC_50_ value of the standard (l-ascorbic acid) was 30.42 µg/mL. The antioxidant activity of the *E. parviflora* extracts may be attributed to the presence of phenolics and flavonoids (Table 2). The variation in the antioxidant potential of different extracts of *E. parviflora* may be due to significant levels of variation in the phenols and flavonoid contents. Besides, the extracting solvent also affects the antiradical scavenging activity of the extract^14^. Among all extracts, the maximum DPPH radical scavenging activity was exhibited by methanolic extract. It can be inferred from the results that methanol could act as the most suitable solvent for the attainment of antioxidant compounds^14^. The extract of methanol has the highest radical removing qualities serving to donate hydrogen atoms or electrons in the DPPH test. These findings proposed that the antioxidant activity of the methanolic extract is due to the high level of phenol and flavonoid compounds^28^. Phenolic compounds have free radical scavenging potential^29^ owing to the presence of hydroxyl groups. Flavonoids prevent reactive oxygen synthesis, forming a chelate with trace elements involved in free-radical production, helping in scavenging reactive species and up-regulating and protecting antioxidant defenses^30^.

**Table 6:** Antioxidant activity of various extracts of *Euphorbia parviflora* by using DPPH method.

Sample	Conc. (µg/mL)	% DPPH radical scavenging activity	IC_50_ (µg/mL)
Ascorbic acid	50	60.73 ± 0.08	30.42
	100	78.56 ± 0.06	
	150	90.62 ± 0.05	
Chloroform extract	50	45.57 ± 0.07	64.54
	100	57.65 ± 0.03	
	150	76.48* ± 0.08	
Methanol extract	50	54.61 ± 0.02	46.63
	100	65.5 7 ± 0.04	
	150	82.42* ± 0.02	
Ethyl acetate extract	50	33.61 ± 0.02	83.72
	100	46.57 ± 0.04	
	150	61.42* ± 0.02	

*Conc* concentration; values indicated are mean ± standard deviation; * shows p value less than 0.01when compared to positive control.

### Antimicrobial activity

Due to the increased emergence of multi-drug resistance in microorganisms worldwide, researchers are working on attaining new constituents from natural resources to combat microbial diseases^31^. The plants are, no doubt, a valuable resource of bioactive constituents posing important medicinal value^17^. In this study, the antimicrobial potential of *E. parviflora* has been analyzed against gram-positive and gram-negative bacteria and fungi (Table 7). The results of antimicrobial activity showed that the methanol extract of *E. parviflora* showed a high (24 ± 0.31) zone of inhibition against *B. subtilis* and *Staphylococcus aureus* while the chloroform extract showed a high zone of inhibition (22 ± 0.40) against *B. subtilis* at 40 mg/mL. However, the ethyl acetate extract showed a high zone of inhibition (18 ± 0.26) against *Klebsiella pneumonia* at 40 mg/mL. The results of antifungal activity showed that the chloroform extract showed a high (26 ± 0.65) zone of inhibition against *Aspergillus niger* while methanol extract showed a high zone of inhibition (25 ± 0.44) against *Candida albicans* at 40 mg/mL (Table 7). However, the ethyl acetate extract showed a high zone of inhibition (21 ± 0.31) against *Aspergillus niger.* Amongst all the extracts the highest antibacterial activity was shown by methanol against *B. subtilis* and *Staphylococcus aureus* as compared to other extracts. This study revealed that Gram-positive bacteria (*B. subtilis* and *Staphylococcus aureus*) were more susceptible to plant extracts as compared to gram-negative bacteria (*E. coli, K. pneumonia, Pseudomonas aeruginosa*). This susceptibility of gram-positive type of bacteria may be due to structural differences in the cell walls of these classes of bacteria. Cells of Gram-negative bacteria possess an extra outer membrane, which provides them with a hydrophilic surface that performs as a permeability barrier for several substances including biological compounds^32^. The antimicrobial activity of plant extracts of *E. parviflora* might be due to several phytochemicals such as phenols, flavonoids, saponins, alkaloids, and tannins (Table 7). Phenols affect the function of the cytoplasmic membrane, disturbing the metabolism of energy, and thus affecting the synthesis of nucleic acids^33^. Terpenoids and alkaloids interact with enzymes and proteins of the microbial cell membrane causing its disruption to disperse a flux of protons towards the cell exterior which induces cell death or may inhibit enzymes necessary for amino acids biosynthesis^34^. Flavonoids have been shown to inhibit bacterial DNA polymerase, RNA polymerase, Reverse Transcriptase, and Telomerase^35^. The saponins decrease surface tension causing an increase in permeability or leakage of cells, resulting in the discharge of intracellular compounds^20^.

**Table 7:** Antimicrobial activity of various extracts of *Euphorbia parviflora.*

Test organisms	Inhibition zone in mm										
	+Ve control	−Ve control	Extract of chloroform in (mg/mL)			Extract of methanol in (mg/mL)			Extract of ethyl acetate in (mg/mL)		
			20	30	40	20	30	40	20	30	40
*Pseudomonas aeruginosa*	28 ± 0.72	0	8 ± 0.53	11 ± 0.40	18 ± 0.33	8 ± 0.63	13 ± 0.28	19 ± 0.51	7 ± 0.19	10 ± 0.30	14 ± 0.21
*Escherichia coli*	26 ± 0.63	0	7 ± 0.40	9 ± 0.34	13 ± 0.45	11 ± 0.51	15 ± 0.23	20 ± 0.32	11 ± 0.60	13 ± 0.23	15 ± 0.37
*Bacillus subtilis*	31 ± 0.54	0	9 ± 0.33	11 ± 0.47	22 ± 0.54	10 ± 0.18	15 ± 0.42	24 ± 0.31	10 ± 0.46	12 ± 0.48	16 ± 0.42
*Staphylococcus aureus*	28 ± 048	0	7 ± 0.42	10 ± 0.50	18 ± 0.40	9 ± 0.53	15 ± 0.52	24 ± 0.56	7 ± 0.20	11 ± 0.45	17 ± 0.19
*Klebsiella pneumonia*	26 ± 68	0	9 ± 0.54	12 ± 0.53	19 ± 0.57	8 ± 0.40	10 ± 0.53	1 5 ± 0.56	8 ± 0.21	13 ± 0.33	1 8 ± 0.26
*Candida albicans*	30 ± 47	0	12 ± 0.66	15 ± 0.60	24 ± 0.45	13 ± 0.52	16 ± 0.62	26 ± 0.44	11 ± 0.52	14 ± 0.62	20 ± 0.44
*Aspergillus niger*	30 ± 36	0	14 ± 0.46	18 ± 0.32	26 ± 0.65	9 ± 0.42	13 ± 0.52	23 ± 0.24	8 ± 0.32	12 ± 0.22	2 1 ± 0.31

The values represent means ± standard deviation, analysis was done using One-way ANOVA followed by Tukey’s test (p ≤ 0.05) as significant.

The minimum inhibitory concentration (MIC) can be termed as the least concentration of antimicrobial agent that can stop the visible increase in the growth of microorganisms after an incubation overnight^21^. The MIC is used to evaluate the antimicrobial effectiveness of new compounds or extracts by measuring the effect of decreasing the antimicrobial concentration. Antimicrobials with low MIC values will be more effective and vice versa. The MIC value of chloroform, methanol, and ethyl acetate extracts of *E. parviflora* ranged from 2 to 6 μg/mL. The lowest MIC values (2) were shown by methanol and chloroform extract against *Bacillus subtilis*, *Staphylococcus aureus*, *and Candida albicans* (Table 8). The values of MIC attained from this research study showed that the methanolic extract and chloroform of *E. parviflora* were more potent against *Bacillus subtilis*, *Staphylococcus aureus*, *and candida albicans*. The differences in bacterial susceptibility may be attributed to the difference in intrinsic tolerance of microorganisms, or the physico-chemical properties of phytochemicals found in the crude extracts of the plant sample^22^.

**Table 8:** MIC values of chloroformic, methanol and ethyl acetate extract of *Euphorbia parviflora.*

MIC values (mg/mL)			
Test organisms	Chloroform extract	Methanol extract	Ethyl acetate extract
*Pseudomonas aeruginosa*	6 ± 0.45	4 ± 0.56	3 ± 0.36
*Escherichia coli*	5 ± 0.47	5 ± 0.40	4 ± 0.45
*Bacillus subtilis*	2 ± 0.60	2 ± 0.44	6 ± 0.50
*Staphylococcus aureus*	2 ± 0.52	2 ± 0.50	5 ± 0.54
*Klebsiella pneumonia*	4 ± 0.50	3 ± 0.60	3 ± 0.33
*Candida albicans*	2 ± 0.37	2 ± 0.63	6 ± 0.62
*Aspergillus niger*	3 ± 0.22	4 ± 0.43	4 ± 0.31

*MIC* minimum inhibitory concentration.

The values represent mean ± standard deviation.

## Conclusion

The efforts of researchers to find plants (natural resources) that might possess antimicrobial potential and also act as an antioxidant are increasing day by day due to the prevalence of a large number of infectious diseases. The extracts obtained from *Euphorbia parviflora* showed the presence of a high value of phenolic and flavonoid contents that may be responsible for the positive effect of the plant in exhibiting strong antimicrobial and antioxidant potential. Further studies are needed to isolate the bioactive compounds from the extract and to elucidate the exact mechanism of action for their possible role in the treatment of several ailments.

## Data Availability

All the data is available in the main manuscript.
