# Publisher Correction: Phytochemical screening, HPLC analysis, antimicrobial and antioxidant effect of *Euphorbia parviflora* L. (Euphorbiaceae Juss.)

**DOI:** 10.1038/s41598-024-59803-z

**Published:** 2024-04-18

**Authors:** Muhammad Adil, Faten Zubair Filimban, Ambrin, Atifa Quddoos, Ayaz Ali Sher, Muhammad Naseer

**Affiliations:** 1 Department of Chemical and Life Sciences, Qurtuba University of Science and Information Technology, Peshawar, Pakistan; 2 Division of Botany, Department of Biological Sciences, Faculty of Science, King Abdulaziz University, Jeddah, Saudi Arabia; 3 Department of Botany, Islamia College, Peshawar, Pakistan; 4 Centre for Plant Sciences and Biodiversity, University of Swat, Charbagh, Pakistan

Correction to: *Scientific Reports* 10.1038/s41598-024-55905-w, published online 07 March 2024

The original version of this Article contained an error in the Funding section where the Grant number was incorrect.

“This work was supported by The Deanship of Scientific Research (DSR) at King Abdulaziz University, Jeddah, Grant No. (No. 1366-247-1443). The authors are highly grateful for this support.”

now reads:

“This work was supported by The Deanship of Scientific Research (DSR) at King Abdulaziz University, Jeddah, Grant No. (IFPRC-187-130-2020). The authors are highly grateful for this support.”

The original Article has been corrected.

