# Author Correction: Phytochemical screening, HPLC analysis, antimicrobial and antioxidant effect of *Euphorbia parviflora* L. (Euphorbiaceae Juss.)

**DOI:** 10.1038/s41598-025-95201-9

**Published:** 2025-04-08

**Authors:** Muhammad Adil, Faten Zubair Filimban, Ambrin, Atifa Quddoos, Ayaz Ali Sher, Muhammad Naseer

**Affiliations:** 1 Department of Chemical and Life Sciences, Qurtuba University of Science and Information Technology, Peshawar, Pakistan; 2 Division of Botany, Department of Biological Sciences, Faculty of Science, King Abdulaziz University, Jeddah, Saudi Arabia; 3 Department of Botany, Islamia College, Peshawar, Pakistan; 4 Centre for Plant Sciences and Biodiversity, University of Swat, Charbagh, Pakistan

Correction to: *Scientific Reports* 10.1038/s41598-024-55905-w, published online 07 March 2024

The original version of this Article contained an error in the Abstract.

“Three samples from methanol, chloroform, and ethyl acetate extract were tested against five different bacterial strains comprising two species from Gram-negative bacteria i.e., *Staphylococcus aureus* and *Bacillus subtilis* and three species from Gram-positive bacteria i.e. *Escherichia coli*, *Pseudomonas aeruginosa* and *Klebsiella pneumonia* along two fungal strains i.e. *Candida albicans* and *Aspergillus niger.*”

now reads:

“Three samples from methanol, chloroform, and ethyl acetate extract were tested against five different bacterial strains comprising two species from Gram-positive bacteria i.e., *Staphylococcus aureus* and *Bacillus subtilis* and three species from Gram-negative bacteria i.e. *Escherichia coli*, *Pseudomonas aeruginosa* and *Klebsiella pneumonia* along two fungal strains i.e. *Candida albicans* and *Aspergillus niger*.”

The original Article has been corrected.

